# Supplementary material for: Multicolor fate mapping of microglia reveals polyclonal proliferation, heterogeneity, and cell-cell interactions after ischemic stroke in mice
Source: Nat Commun. 2025 Sep 16;16:8294. doi: 10.1038/s41467-025-63949-3 (PMC12441135; doi:10.1038/s41467-025-63949-3)
Supplement: Supplementary file 1 — Supplementary Information [file 41467_2025_63949_MOESM1_ESM.pdf]

Multicolor fate mapping of microglia reveals polyclonal proliferation, heterogeneity, and cell-cell interactions after ischemic stroke in mice

List of supplementary materials:

- Supplementary Figures 1-9.
- Supplementary Movies 1-5
- Source data
- Custom code
- Demo data for running the code

Supplementary Fig. 1

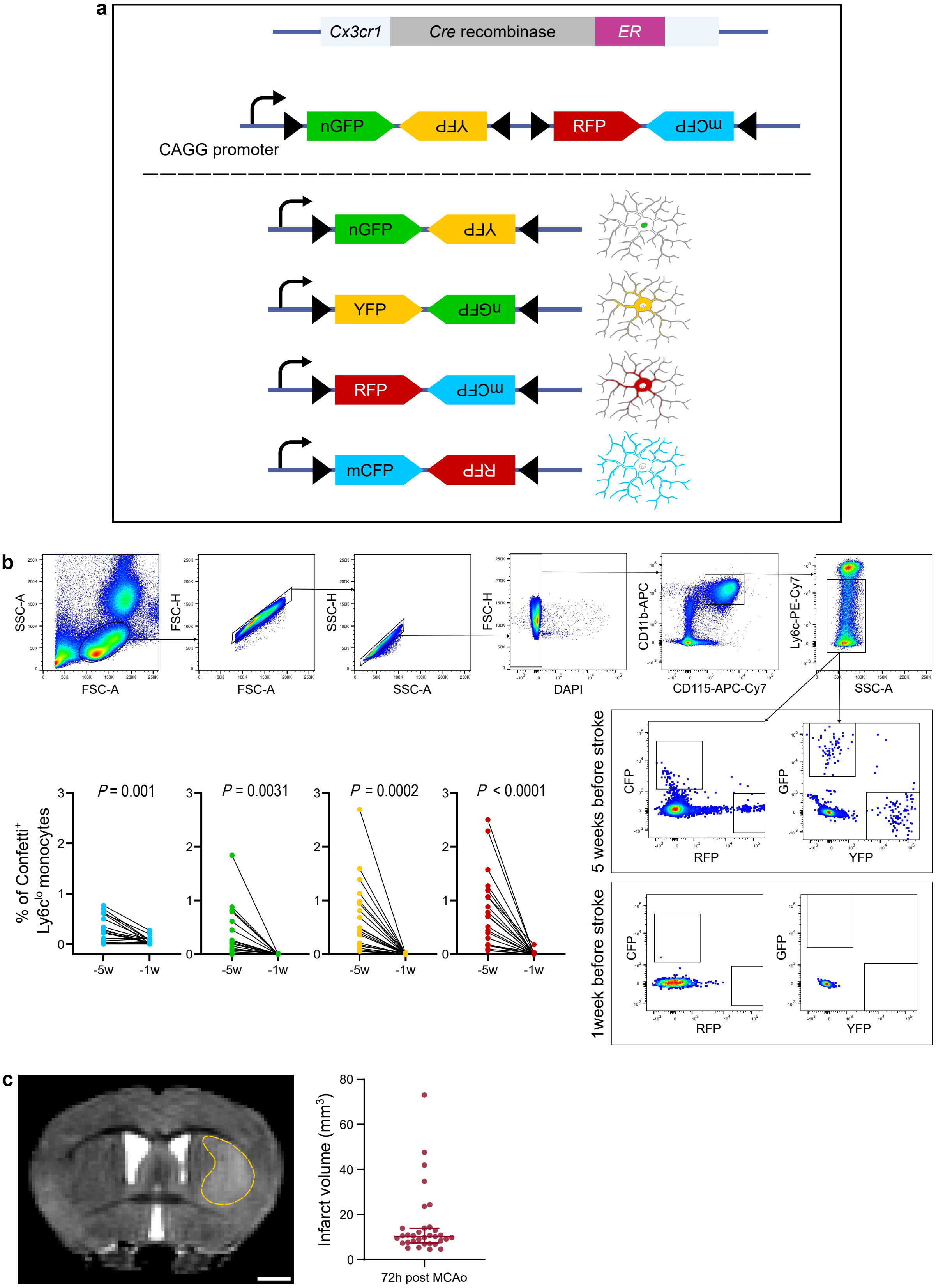

Supplementary Fig. 1: Experimental details and model descriptions

a The genetic structure of the Microfetti mouse, where the fractalkine receptor gene *Cx3cr1* is replaced with a Cre recombinase fused to a mutant estrogen ligand-binding domain ($Cx3cr1^{CreER}$). In addition, these mice express the Confetti structure in the *Rosa 26* locus together with the strong CAGG promoter and a LoxP-flanked Neo^R-cassette. Upon tamoxifen injection and Cre-mediated recombination, one of four fluorescent proteins, e.g., nuclear GFP (nGFP, green), cytoplasmic YFP (yellow), cytoplasmic RFP (red), or membrane-tagged CFP (mCFP, cyan), is stochastically expressed in $Cx3cr1^+$ cells. Created in BioRender. Göttert, R. (2025) https://BioRender.com/xdhxpif **b** Flow cytometry analysis of blood samples from Microfetti mice, one and five weeks before stroke. $Ly6c^{lo}$ monocytes expressed the Confetti markers one week after the tamoxifen injection. However, due to their short lifespan, these cells are absent after four weeks. This ensures that the $Confetti^+$ cells detected in the brain after MCAo are not derived from invading monocytes. Statistical analysis with paired two-tailed t test. Number of animals $N = 21$. c Representative T2-weighted MRI image 72 h after MCAo showing a typical stroke lesion in the dorsolateral striatum (caudoputamen). Scale bar: 1 mm. Infarct volumes based on MR-imaging. Data presented as median ± IQR. $N = 33$ (of 43 mice used for the analyses in Fig. 1 and Fig. 2. Mice sacrificed 2 days after MCAo did not receive an MRI measurement, $N = 8$. Two mice had to be excluded because of insufficient MR-image quality for accurate measurement of lesion volume.)

Supplementary Fig. 2

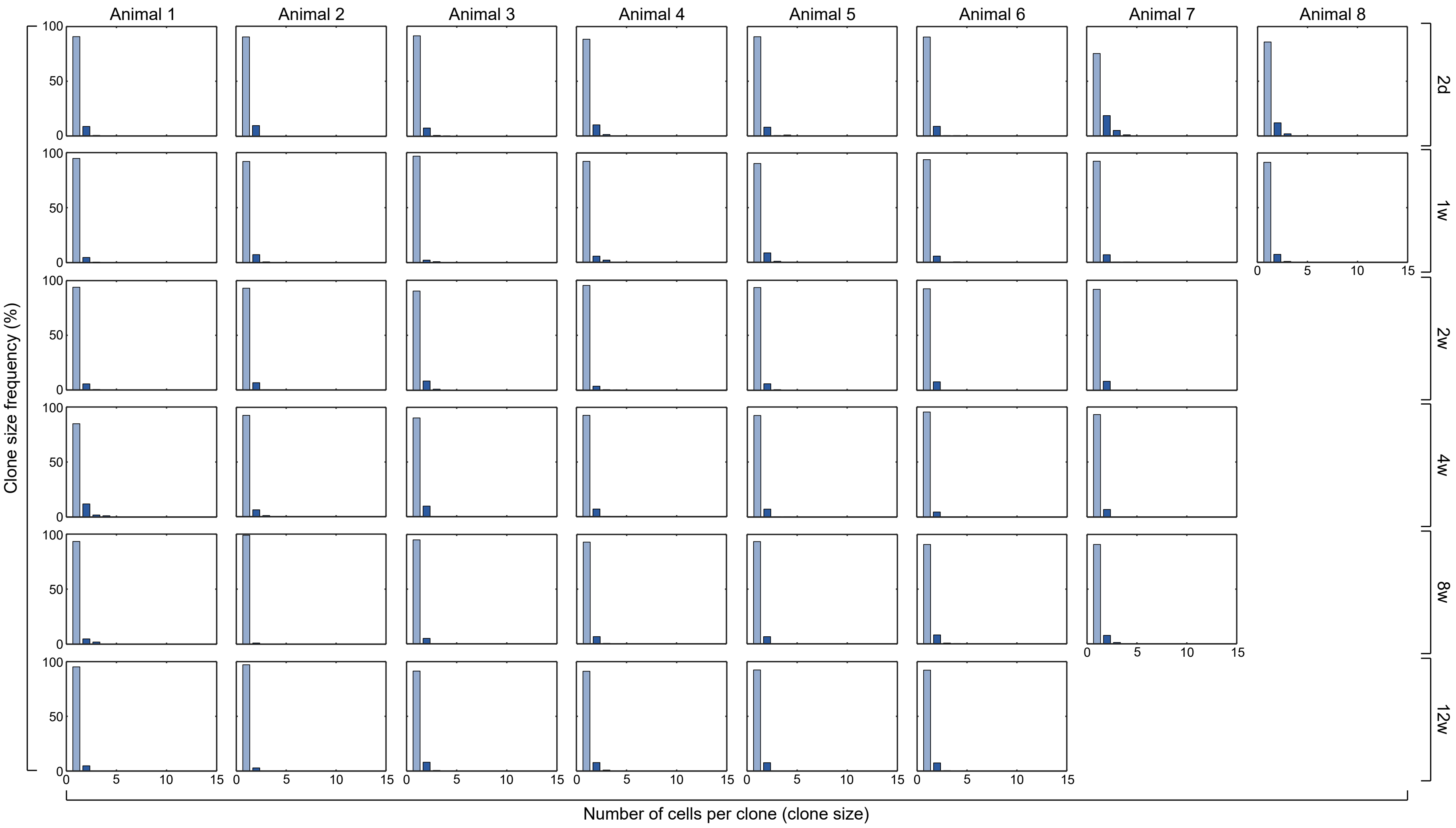

Supplementary Fig. 2: Distribution of clone size based on spatial clustering with DBSCAN in the contralateral nonischemic dorsolateral striatum

Histograms of clone sizes in the contralateral striatum from individual mice at all time points. Singlets are represented in light blue. The data show that in almost all mice, 90-100% of Confetti^{+} cells are present as singlets with very few occasional small clones of 2-3 cells.

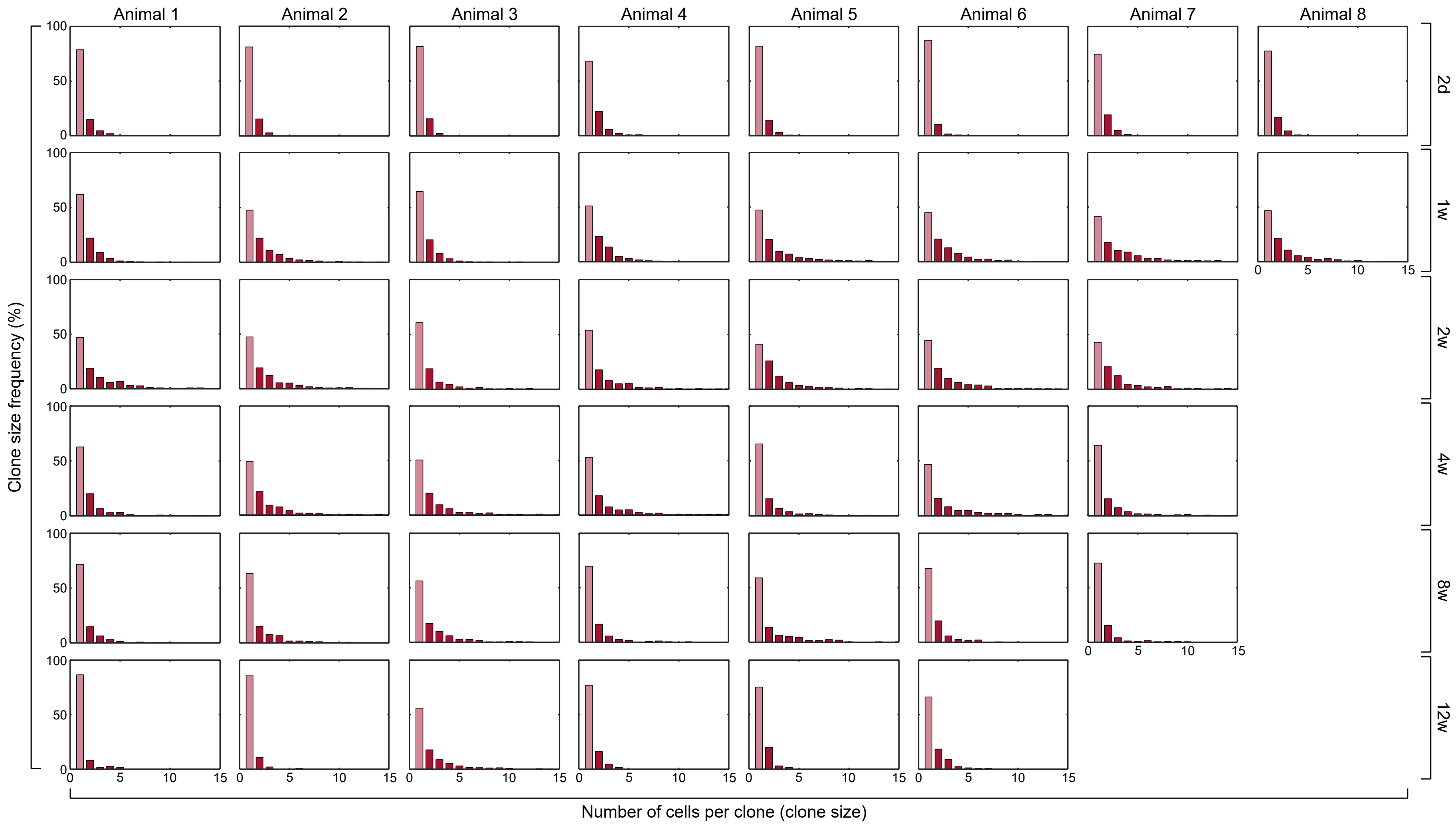

Supplementary Fig. 3: Distribution of clone size based on spatial clustering with DBSCAN in the ischemic dorsolateral striatum

Histograms of clone sizes in the ischemic dorsolateral striatum from individual mice at all time points. Singlets are represented in light red color. The data show that small clones are much more frequent than larger clones. In other words, the frequency of a specific clone size decreases exponentially with the clone size. Please note that there are many clones larger than the upper limit on the x-axis (15 cells). However, the frequency of these events is much smaller than the frequency of smaller clones, making the visualization of those events challenging.

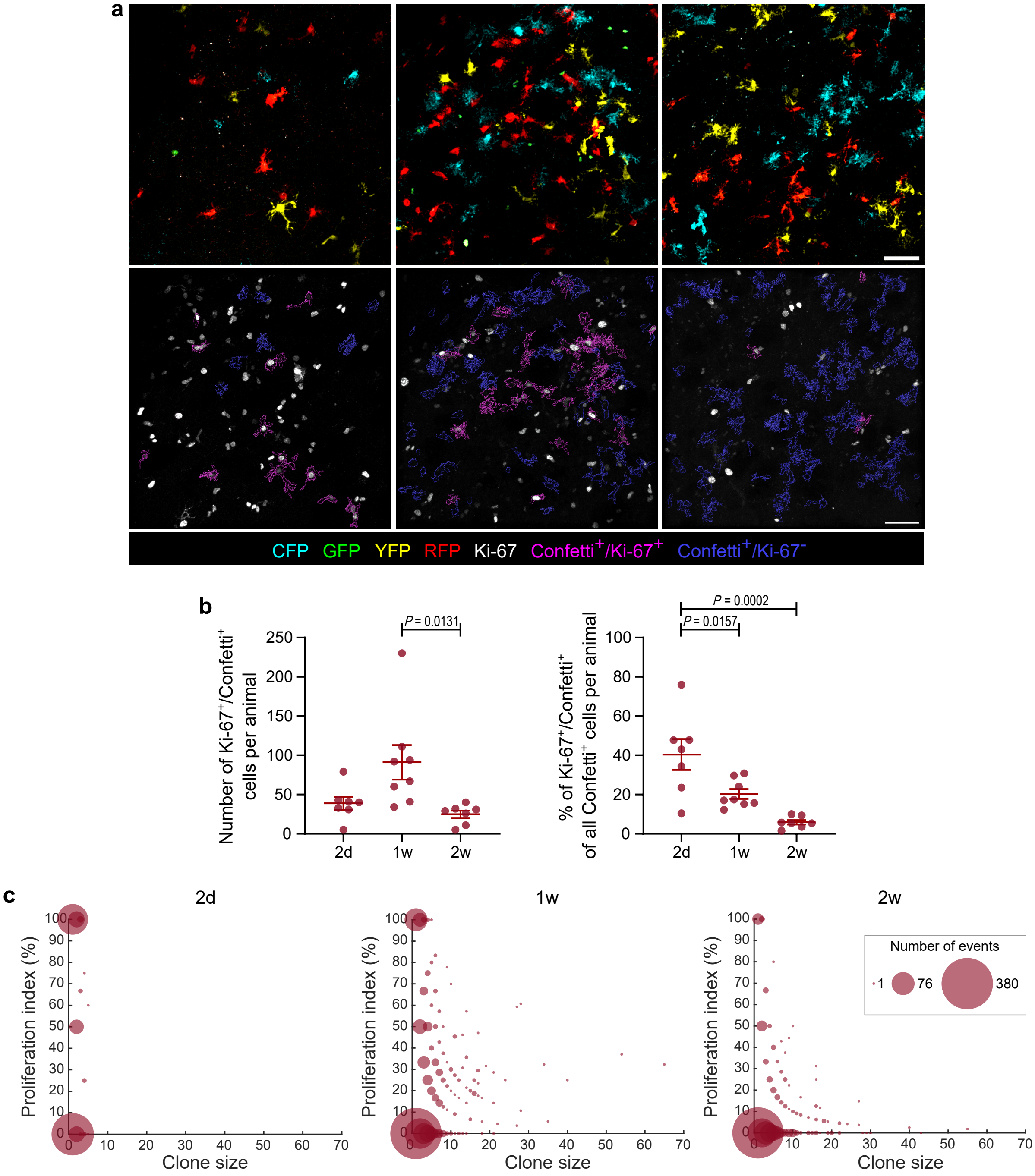

Supplementary Fig. 4: Microglial proliferation dynamics after ischemic stroke using Ki-67 analysis combined with DBSCAN clustering

a Representative examples of fluorescent images from the ipsilateral striatum of Microfetti mice at 2d, 1w, and 2w after stroke. The first row shows the Confetti markers, while the second row shows surface renderings of the Confetti cells and their classification as Ki-67$^+$ (in magenta) and Ki-67$^-$ (in blue) based on Ki-67 immunohistochemistry (in white). Scale bars: 50 µm. **b** Comparison of the total number of Ki-67$^+$ cells and the percentage of Ki-67$^+$ of all Confetti$^+$ cells per animal. Means ± s.e.m. are shown. Statistical analysis was performed with one-way ANOVA followed by Tukey's test for multiple comparisons. Number of animals N = 7 (2d), 8 (1w), 7 (2w). **c** Proliferation index analysis for single clones at the three time points. A DBSCAN analysis was executed similar to that shown in Fig. 2, and then a proliferation index (number of Ki-67$^+$ cells in one clone × 100 / clone size) was calculated for each clone. The size of the circle represents the number of events (clones or singlets) that share similar values (proliferation index and clone size).

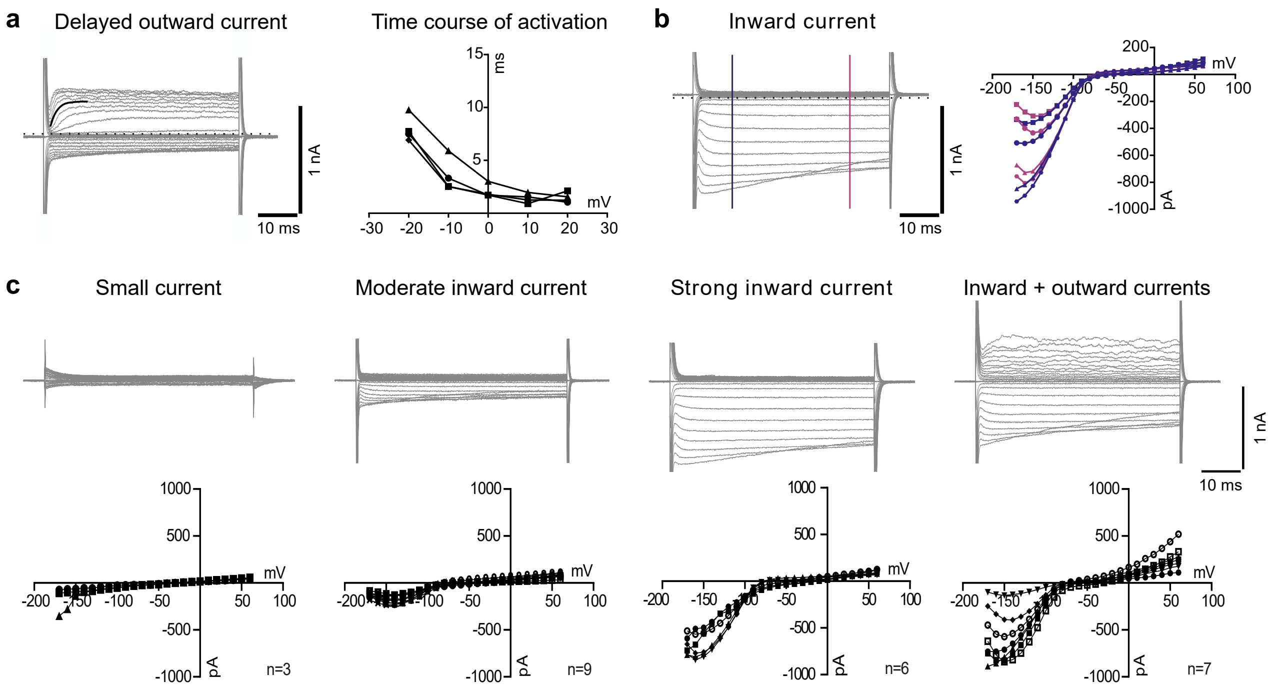

Supplementary Fig. 5: Characterization of inward and delayed outward currents of microglia and depiction of different groups of currents 8 weeks after MCAo.

a Exemplary trace of delayed outward current (holding potential -70 mV) and analysis of 4 exemplary traces (from 2 and 7 days after MCAo) with respect to current kinetics. The time course of activation (Tau) was determined by an exponential fit over a delayed incline and showed voltage-dependent characteristics. **b** Exemplary trace of inward current (holding potential -20 mV) and IV-curves of 4 exemplary traces. The blue and purple traces represent values recorded 10 and 40 ms after the initiation of the pulse (first and second line), respectively. This indicates a voltage-dependent inactivation of the inward current. **c** 8 weeks after MCAo, microglial cells within the ischemic tissue exhibited significant heterogeneity, manifesting diverse current patterns. These current patterns could be categorized into four groups: cells with small currents (comparable to control cells), cells with moderate inward current but no outward current, cells with very strong inward current but no outward current, and cells with inward and (not delayed) outward currents. The groups are represented by exemplary traces and IV-curves (holding potential -70 mV).

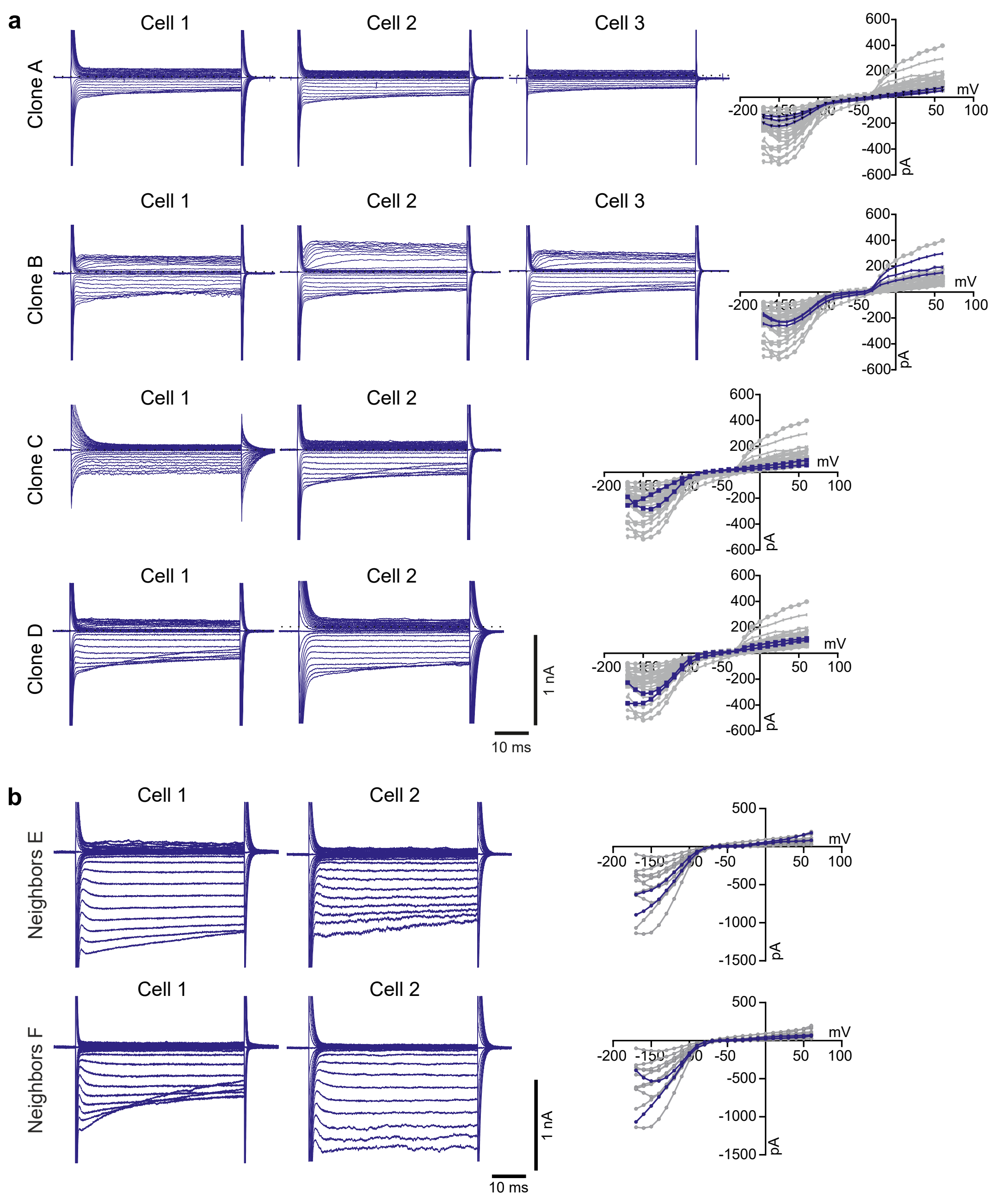

Supplementary Fig. 6: Similarity of microglial membrane properties within clones 1 week after stroke, with additional recordings from neighboring cells after 8 weeks.

a Whole-cell patch-clamp recordings from different clones 1 week after MCAo. Each row consists of 2-3 raw traces and IV-curves from different cells of one clone. The IV-curves show the presented traces in blue against all other cells from the same time point in gray. **b** Recordings from two pairs of neighboring cells from different clones 8 weeks after MCAo (extended data of Fig. 4c).

Supplementary Fig. 7

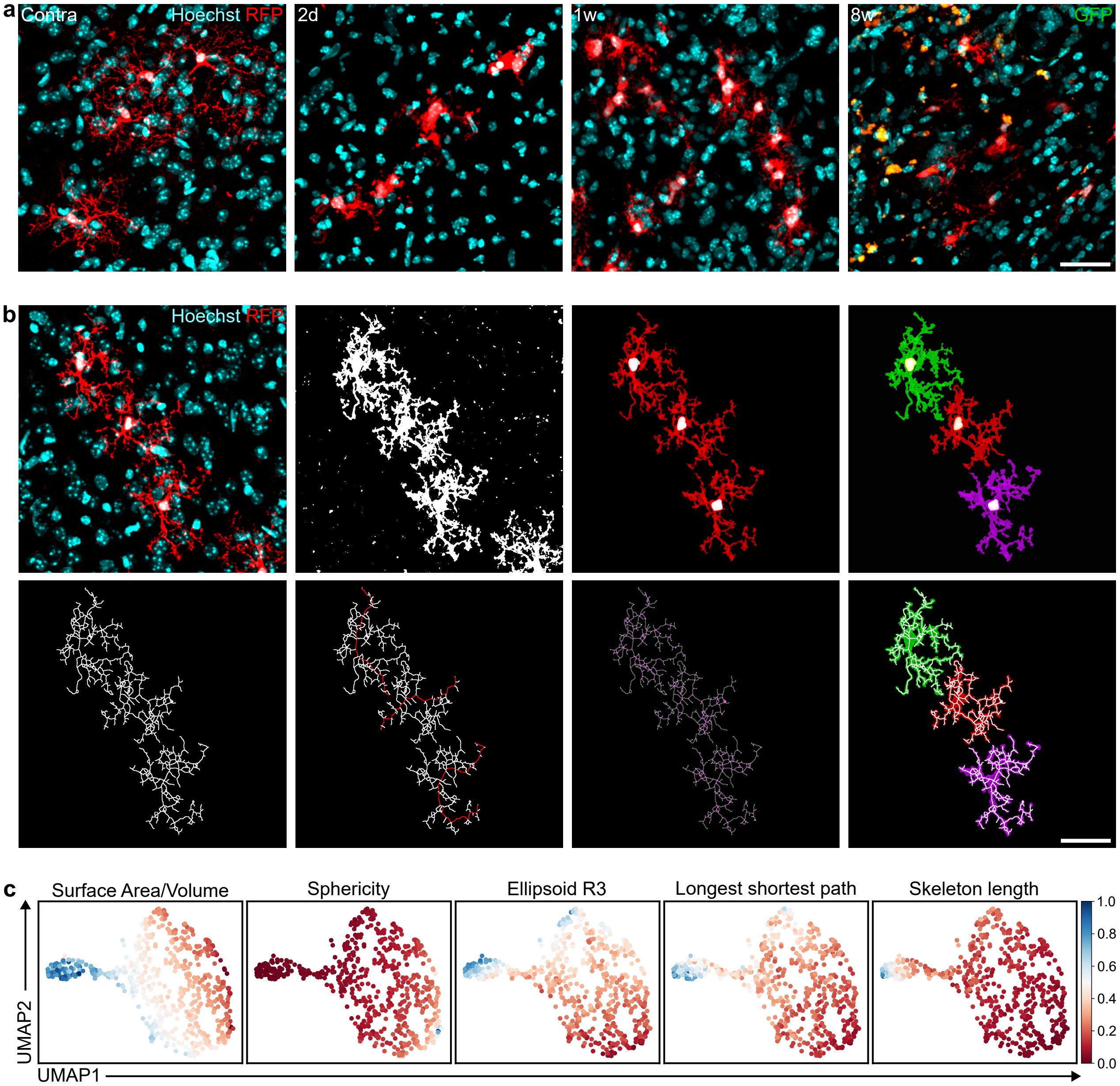

Supplementary Fig. 7: The pipeline of the automated morphology analysis of microglia.

a Fluorescent images of RFP$^+$ microglia corresponding to the 3D renderings shown in (Fig. 5a). Scale bar: 30 µm. **b** The morphological analysis pipeline including the following steps: (1) fluorescent image of RFP$^+$ microglia and Hoechst nuclear staining, (2) binary results of image segmentation with U-Net of the RFP$^+$ channel, (3) detection of nuclei within the RFP$^+$ cells and exclusion of cells that cross the image border, (4) isolation of single cells using the nuclei as seeds for water-shedding,(5) binary skeletonizing, (6) detection of the longest shortest path, (7) detection of junctions and end-point voxels, and (8) overlaying of the cellular skeletons and the cell volumes. Scale bar: 30 µm. **c** UMAP plots based on the morphological parameters of 668 cells from the ischemic and contralateral striatum at 2 days, 1 week, and 8 weeks. For each plot, the color spectrum represents the values of one morphological parameter.

Supplementary Fig. 8

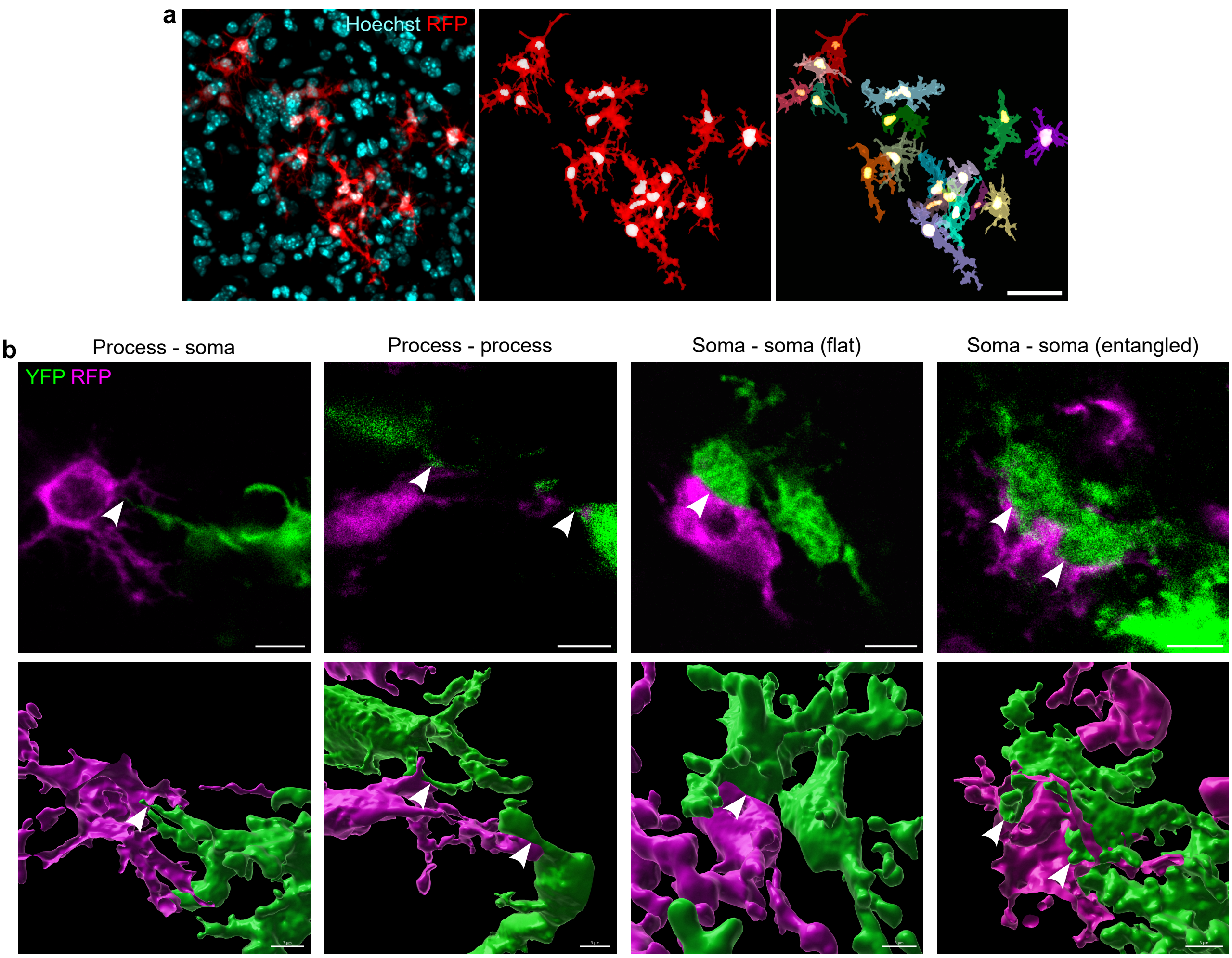

Supplementary Fig. 8: Microglial cell-cell interactions after ischemic stroke

a Example of RFP^+ microglial cells appearing in close contact with each other in the ischemic striatum one week after MCAo. Fluorescent image, 3D rendering with all cells in red and their nuclei in white, and 3D rendering with each cell in a different color. Scale bar: 30 µm. **b** Different types of cell-cell inter-clonal interactions of microglia after stroke depicted by conventional confocal laser scanning microscopy. The upper row shows single *z*-plane images, and the lower row shows 3D renderings. White arrowheads indicate points of contact between cells. Scale bars: 5 µm in the upper row and 3 µm in the lower row.

Supplementary Fig. 9

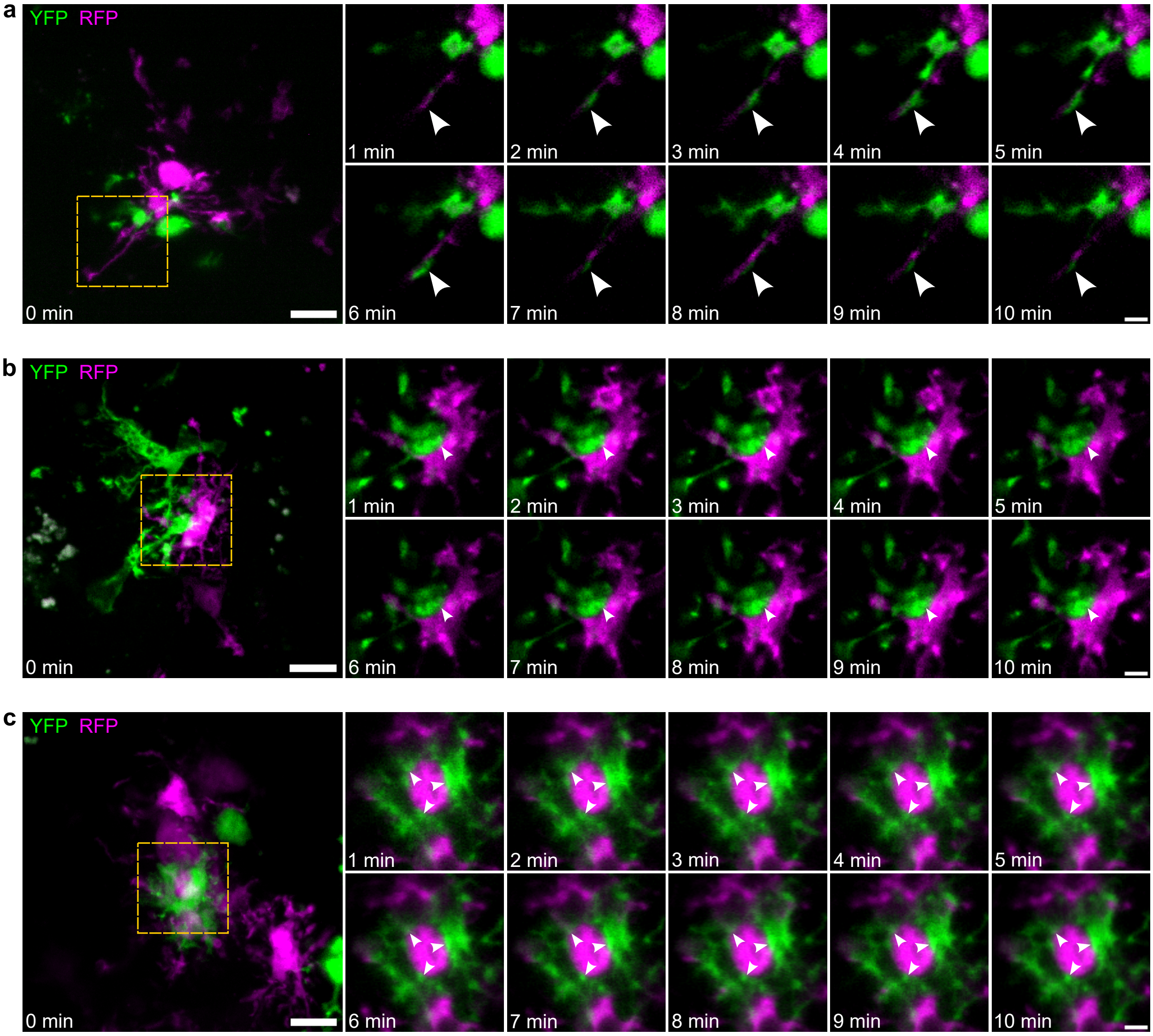

Supplementary Fig. 9: Dynamics of inter-clonal cell-cell interactions of microglia after ischemic stroke revealed by live-cell imaging of acute brain slices

a Dynamic process-process microglial interaction, where a YFP^{+} cell (in green) extends a process to wrap around and slide over a process of an RFP^{+} cell (in magenta). The time-lapse shows how the YFP^{+} process dynamically slides over the stationary RFP^{+} process (see Supplementary Movie 3). **b** Two microglial cells showing a soma-soma interaction with little entanglement. Despite the dynamic movements of the cells' processes, the interaction remains stable for the duration of the 10-minute recording (see Supplementary Movie 4). **c** Multiple YFP^{+} and RFP^{+} microglial cells are entangled in one nodule, exhibiting several interaction points between multiple cells. The nodule is stable over the duration of the 15-minute recording (see Supplementary Movie 5). In each panel, the left image corresponds to an MIP, while the enlarged images show a single *z*-plane over a 10-minute recording period. White arrowheads indicate points of contact between cells. Scale bars: 10 µm for the left images and 3 µm for the enlarged images.
