# Supplementary material for: Multicolor fate mapping of microglia reveals polyclonal proliferation, heterogeneity, and cell-cell interactions after ischemic stroke in mice
Source: Nat Commun. 2025 Sep 16;16:8294. doi: 10.1038/s41467-025-63949-3 (PMC12441135; doi:10.1038/s41467-025-63949-3)
Supplement: Supplementary file 2 — Description of Additional Supplementary Files [file 41467_2025_63949_MOESM2_ESM.pdf]

Description of Additional Supplementary Files

Supplementary Movie 1: 3D rendering of microglial cell-cell interaction related to Fig. 6a.

Supplementary Movie 2: Live-cell imaging of microglial process-soma interaction in acute brain slices. Related to Fig. 6c.

Supplementary Movie 3: Live-cell imaging of microglial process-process interaction in acute brain slices. Related to Supplementary Fig. 9a.

Supplementary Movie 4: Live-cell imaging of microglial flat soma-soma interaction in acute brain slices. Related to Supplementary Fig. 9b.

Supplementary Movie 5: Live-cell imaging of microglial entangled soma-soma interactions in acute brain slices. Related to Supplementary Fig. 9c.
