# Supplementary material for: Multicolor fate mapping of microglia reveals polyclonal proliferation, heterogeneity, and cell-cell interactions after ischemic stroke in mice
Source: Nat Commun. 2025 Sep 16;16:8294. doi: 10.1038/s41467-025-63949-3 (PMC12441135; doi:10.1038/s41467-025-63949-3)
Supplement: Supplementary file 8 — Reporting Summary [file 41467_2025_63949_MOESM8_ESM.pdf]

nature portfolio

Corresponding author(s): Karen Gertz

Last updated by author(s): 18.08.2025

Reporting Summary

Nature Portfolio wishes to improve the reproducibility of the work that we publish. This form provides structure for consistency and transparency in reporting. For further information on Nature Portfolio policies, see our Editorial Policies and the Editorial Policy Checklist.

Statistics

For all statistical analyses, confirm that the following items are present in the figure legend, table legend, main text, or Methods section.

n/a	Confirmed	
☐	☒	The exact sample size (n) for each experimental group/condition, given as a discrete number and unit of measurement
☐	☒	A statement on whether measurements were taken from distinct samples or whether the same sample was measured repeatedly
☐	☒	The statistical test(s) used AND whether they are one- or two-sided<br>*Only common tests should be described solely by name; describe more complex techniques in the Methods section.*
☒	☐	A description of all covariates tested
☐	☒	A description of any assumptions or corrections, such as tests of normality and adjustment for multiple comparisons
☐	☒	A full description of the statistical parameters including central tendency (e.g. means) or other basic estimates (e.g. regression coefficient) AND variation (e.g. standard deviation) or associated estimates of uncertainty (e.g. confidence intervals)
☐	☒	For null hypothesis testing, the test statistic (e.g. F, t, r) with confidence intervals, effect sizes, degrees of freedom and P value noted<br>*Give P values as exact values whenever suitable.*
☒	☐	For Bayesian analysis, information on the choice of priors and Markov chain Monte Carlo settings
☒	☐	For hierarchical and complex designs, identification of the appropriate level for tests and full reporting of outcomes
☒	☐	Estimates of effect sizes (e.g. Cohen's d, Pearson's r), indicating how they were calculated

Our web collection on statistics for biologists contains articles on many of the points above.

Software and code

Policy information about availability of computer code

Data collection	Data was collected using the following software: ZEN 2.3.64.0 (black edition), LAS AF 2.7.9723.3, BD FACSDiva 8.0.2, Bruker Software Paravision 5.1, TIDA 5.24, NIS-Elements AR 5.42.06 (Build 1821), Abberior LiGHTBOX 2023.26.18334-win64.
Data analysis	Statistics were run through Prism - GraphPad software (Version 9). MATLAB R2022a was used to perform Monte Carlo simulation analysis and cluster analysis with the following toolboxes: Statistics and Machine Learning Toolbox, Antenna Toolbox™, and Parallel Computing Toolbox. FACS data were analyzed by FlowJo software v10.6.1. For image analysis, Imaris software v9.7, or Fiji software v1.54f and its plugins LabKit, MorphoLibJ, 3D Suite were used. The following Python (3.9) packages were used for morphology analysis: Jupyter Notebook 7.0.6, TensorFlow-GPU 2.11.0, Keras 2.11.0, NumPy 1.24.2, patchify 0.2.3, glob2 0.7, czifile 2019.7.2, tifffile 2023.2.28, Matplotlib 3.7.0, Pandas 2.1.4, seaborn 0.13.2, pingouin 0.5.4, scikit_posthocs 0.8.1, ipywidgets 8.1.1, scikit-learn 1.6.1, umap-learn 0.5.7. MRI data was analyzed by Analyze v10.0. Electrophysiological data were analyzed with Igor Pro 6.37. Final figures were produced using Inkscape software v1.3 and Adobe Illustrator 2025.<br>All custom code needed to reproduce the analyses presented in this study was made available in this public GitHub repository: https://github.com/kikhiam/kikhia_et_al_2025 (https://doi.org/10.5281/zenodo.16891419)

For manuscripts utilizing custom algorithms or software that are central to the research but not yet described in published literature, software must be made available to editors and reviewers. We strongly encourage code deposition in a community repository (e.g. GitHub). See the Nature Portfolio guidelines for submitting code & software for further information.

Data

Policy information about availability of data

All manuscripts must include a data availability statement. This statement should provide the following information, where applicable:

- Accession codes, unique identifiers, or web links for publicly available datasets
- A description of any restrictions on data availability
- For clinical datasets or third party data, please ensure that the statement adheres to our policy

The authors declare that the data supporting the findings of this study are provided within the manuscript and the supplementary materials. Source data are provided with this paper. For running the code and reproducing analyses, additional data were made publicly available here: https://doi.org/10.17605/OSF.IO/H9FJY

Research involving human participants, their data, or biological material

Policy information about studies with human participants or human data. See also policy information about sex, gender (identity/presentation), and sexual orientation and race, ethnicity and racism.

Reporting on sex and gender	N/A
Reporting on race, ethnicity, or other socially relevant groupings	N/A
Population characteristics	N/A
Recruitment	N/A
Ethics oversight	N/A

Note that full information on the approval of the study protocol must also be provided in the manuscript.

Field-specific reporting

Please select the one below that is the best fit for your research. If you are not sure, read the appropriate sections before making your selection.

☒ Life sciences ☐ Behavioural & social sciences ☐ Ecological, evolutionary & environmental sciences

For a reference copy of the document with all sections, see nature.com/documents/nr-reporting-summary-flat.pdf

Life sciences study design

All studies must disclose on these points even when the disclosure is negative.

Sample size	Sample size was pragmatically operated on the basis of the observed biological variability
Data exclusions	Animals were excluded if the cre recombination resulted in an inadequate expression of the Confetti reporters.
Replication	All experiments were confirmed on multiple cohorts of animals. All cohorts were reported in the study.
Randomization	Mice were allocated randomly to the different timepoint groups.
Blinding	Researchers were not blinded during image acquisition, patch-clamp recordings, and blood staining (FACS) because researchers were involved in post-surgery care of mice and, therefore, had knowledge about reperfusion timepoints.

Reporting for specific materials, systems and methods

We require information from authors about some types of materials, experimental systems and methods used in many studies. Here, indicate whether each material, system or method listed is relevant to your study. If you are not sure if a list item applies to your research, read the appropriate section before selecting a response.

Materials & experimental systems

n/a	Involved in the study
[ ]	[x] Antibodies
[x]	[ ] Eukaryotic cell lines
[x]	[ ] Palaeontology and archaeology
[ ]	[x] Animals and other organisms
[x]	[ ] Clinical data
[x]	[ ] Dual use research of concern
[x]	[ ] Plants

Methods

n/a	Involved in the study
[x]	[ ] ChIP-seq
[ ]	[x] Flow cytometry
[x]	[ ] MRI-based neuroimaging

Antibodies

Antibodies used

anti-Iba-1 antibody (rabbit, Fujifilm Wako, 019-19741), anti-P2RY12 (rabbit, 1:250, AnaSpec, AS-55043A), Alexa Fluor 647 (Invitrogen, A31573), anti-Ki-67 (rat, 1:500, eBioscience™, 14-5698-82), STAR RED (goat anti-rabbit IgG, 1:200, abberior, STRED-1002-500UG), CD45-BV711 (BioLegend, 109847), CD11b-APC (BioLegend, 101212), CD115-APC-Cy7, (BioLegend, 135531), Ly6-PE-Cy7 (BioLegend, 128018).

Validation

All antibodies used in this study are commercially available and have been validated by manufacturers and numerous previous studies:
https://labchem-wako.fujifilm.com/us/category/01213.html
https://www.hoelzel-biotech.com/en/anaspec-antibody-other-as-55043a-anti-p2y12-mouse.html
https://www.thermofisher.com/antibody/product/Ki-67-Antibody-clone-SolA15-Monoclonal/14-5698-82
https://www.thermofisher.com/antibody/product/Donkey-anti-Rabbit-IgG-H-L-Highly-Cross-Adsorbed-Secondary-Antibody-Polyclonal/A-31573
https://abberior.shop/abberior-STAR-RED-goat-anti-rabbit-IgG-500-ll-1-mg-ml
https://www.biolegend.com/fr-lu/products/brilliant-violet-711-anti-mouse-cd45-2-antibody-13278?GroupID=BLG1934
https://www.biolegend.com/fr-lu/products/apc-anti-mouse-human-cd11b-antibody-345
https://www.biolegend.com/fr-lu/products/apc-cyanine7-anti-mouse-cd115-csf-1r-antibody-13759
https://www.biolegend.com/fr-lu/products/pe-cyanine7-anti-mouse-ly-6c-antibody-6063

Animals and other research organisms

Policy information about studies involving animals; ARRIVE guidelines recommended for reporting animal research, and Sex and Gender in Research

Laboratory animals

Adult female Cx3cr1creER/+R26RConfetti/+ mice were used for this study.

Wild animals

No wild animals were used in the study.

Reporting on sex

Only female mice were used in the study in accordance to previous studies with same mouse strain.

Field-collected samples

No field-collected samples were used in the study.

Ethics oversight

All experimental procedures were approved by the State Office for Health and Social Affairs in Berlin (LAGeSo) and conducted following the German Animal Welfare Act and Animal Welfare Regulation Governing Experimental Animals (TierSchVersV).

Note that full information on the approval of the study protocol must also be provided in the manuscript.

Plants

Seed stocks

N/A

Novel plant genotypes

N/A

Authentication

N/A

Flow Cytometry

Plots

Confirm that:

- [x] The axis labels state the marker and fluorochrome used (e.g. CD4-FITC).
- [x] The axis scales are clearly visible. Include numbers along axes only for bottom left plot of group (a 'group' is an analysis of identical markers).
- [x] All plots are contour plots with outliers or pseudocolor plots.
- [x] A numerical value for number of cells or percentage (with statistics) is provided.

Methodology

Sample preparation	Blood was collected from the facial vein in capillary blood collection tubes (Microvette® 500 K3 EDTA, 20.1341.100). Within 30 minutes, it was transferred into 5 mL polystyrene tubes (Falcon, 352235) and incubated with rat anti-Mouse CD16/CD32 antibody (BD Biosciences, 431 553141) for 5 min at 4°C. Then antibodies were added and samples were incubated for 30 min at 4°C: C. Afterward, we lysed the red blood cells with BD Pharm Lyse (BD Biosciences, 55589 434 9) according to the manufacturer's instructions. The samples were washed twice and resuspended with FACS buffer (PBS, 0.5% BSA) and analyzed with BD LSR II Flow Cytometer.
Instrument	BD® LSR II Flow Cytometer
Software	Data collection: BD FACSDiva 8.0.2<br>Data analysis: FlowJo software v10.6.1
Cell population abundance	For gating strategy please see Supplementary Fig. 1. Abundance of relevant cell population was between 0% and 3% of confetti+ Ly6Clo monocytes. Minimum of 50,000 white blood cells have been recorded per sample.
Gating strategy	Positive and negative boundaries were determined by the unstained control vs. single staining controls.

- [x] Tick this box to confirm that a figure exemplifying the gating strategy is provided in the Supplementary Information.
